# Strontium Bromide in Epilepsy

**Published:** 1907-03

**Authors:** 


### STRONTIUM BROMIDE IN EPILEPSY.

Bennion has used strontium bromide in the treatment of epilepsy in the insane, and arrives at the following conclusions: Strontium bromide, as a rule, acts better in controlling the number and severity of the fits than the mixed bromides of sodium and potassium. Strontium bromide rarely causes depression. Rashes were absent in all his cases. It acted best and most consistently in the female patients, in all of whom the action of this drug, as regards controlling the fits, was far superior to that of the mixed bromides. The maniacal symptoms of the male patients while taking strontium bromide were not influenced to any perceptible degree. The mental condition of the female patients seemed to improve. Considering the severity of the type of epilepsy observed, it is reasonable to suppose that with a mildr form of epilepsy than is usually met with in an asylum an even greater benefit would be derived from the administration of this drug. All those having epileptics under their care should give the bromide of strontium a fair trial.—Lancet, January 5th, 1907.

